# Threat-responsiveness and the decision to obtain free influenza vaccinations among the older adults in Taiwan

**DOI:** 10.1186/1471-2458-9-275

**Published:** 2009-07-31

**Authors:** Ying-Chun Li, Chi-Mei Liu

**Affiliations:** 1 Institute of Health Care Management, National Sun Yat-Sen University, 70 Lien-Hai Rd., Kaohsiung 804, Taiwan, Republic of China

## Abstract

**Background:**

Although older adults are encouraged by government agencies to receive influenza vaccinations, many do not obtain them. In Taiwan, where universal health care coverage has significantly reduced the barriers of access to care, the health care system has provided free influenza vaccinations for people 65 years or older since 2001. Nevertheless, the numbers of people who use this service are much fewer than expected. The aim of this study was to explore major factors that might affect the decision to receive influenza vaccinations among older adults in Taiwan.

**Methods:**

Using national representative health insurance medical claims from the National Health Insurance Research Database between 2002 and 2004, we investigated the role of threat-responsiveness, represented by prior vaccinations and prior physician visits for flu-like respiratory conditions, in the decisions of older adults to obtain vaccinations in Taiwan.

**Results:**

Among the sample of 23,023 older adults, the overall yearly vaccination rates in this study were 38.6%, 44.3% and 39.3% for 2002, 2003, and 2004, respectively. Adjusting for covariates of individual and health care facility characteristics, the multivariate logistic regression revealed that older adults who had had prior vaccinations were ten times more likely to be vaccinated during the following influenza season than those who had not (OR = 10.22, 95%CI: 9.82–10.64). The greater the frequency of prior physician visits for flu-like respiratory conditions, the greater the likelihood that one would decide to be vaccinated. Visits during prior interim (non-epidemic) season exerted a stronger positive influence than prior influenza season on this likelihood (OR = 1.59, 95% CI: 1.46–1.73 vs. OR = 1.11 95% CI: 1.01–1.22, respectively).

**Conclusion:**

Threat-responsiveness, or perceived risk, greatly influences influenza vaccination rates among the older adults in Taiwan. These findings can be used to help design public health campaigns to increase the influenza vaccination rate in this vulnerable group of citizens. Particularly, older adults who never had influenza vaccinations can be identified, educated, and encouraged to participate.

## Background

Influenza poses a significant threat to the health of older adults and presents a critical disease burden to health care delivery systems around the world [1-3]. While many studies and government health systems consider vaccination to be a cost-effective means of preventing influenza in older adult populations [4-7], vaccination rates do not reach the number of people targeted to receive them in many countries [8-11].

In Taiwan, where universal health care coverage has significantly reduced the barriers to care [12], the health care system has provided free influenza vaccinations for people 65 years or older since 2001. In fact, Taiwan's federal government has set a vaccination rate goal of 68% for older adults by 2010. Although the barriers to preventive services have been reduced and people have been encouraged to use them, the number of people using them are much fewer than expected [13]. Therefore, public health authorities and policy makers may be interested in what other factors may be involved in the decision of an older adult to obtain an influenza vaccination.

Threat-responsiveness is the hypothesis that individuals will alter their prevention behaviors when threat is perceived. Threat-responsiveness can be interpreted as fear of sickness. Therefore, it is assumed the greater the prevalence of disease, the greater the perceived threat and the greater the level of prevention. Threat-responsiveness would be expected to operate in human populations faced with any infectious disease, and has been found, for example, to influence whether a person would take measures to prevent such infectious diseases as influenza, measles, or AIDS [14-17]. Perceived threat has also been found to be predictive of whether older adults would obtain influenza vaccinations [18]. However, few studies have empirically tested the influence of perceived threat on utilization of preventive medicine on a national level. To the best of our knowledge, no such study has been conducted in Taiwan. Using national health insurance claims data, we studied the association between threat-responsiveness, represented by prior influenza vaccinations and prior visits to physicians for flu-like respiratory conditions, and the decision to receive influenza vaccinations during a current epidemic season in Taiwan.

## Methods

We collected demographic and medical information for 200,000 individuals for the years 2002 to 2004 from a random sample of Taiwan's National Health Insurance claims records provided by the National Health Research Institutes. The data sets included demographic and health care history, including outpatient records, inpatient records, and information about the medical facilities they visited during the study period. Of that random sample of 200,000 claimants, there were 23,779 people aged 65 years or older. We excluded 756 persons who had died or whose data were incomplete, leaving us with a final sample of 23,023 older adults whose vaccination and medical care history we tracked throughout the study period. The names of the subjects and the names of the specific medical facilities were unknown to the researchers and personal and facility identification numbers were scrambled, making it unnecessary to obtain informed consent to link the data.

In this study, our proxies for threat-responsiveness were previous vaccinations and previous health care utilization. We assumed that vaccination history or utilization of health care services for flu-like respiratory conditions could indicate whether the receiver perceived some increased threats to his or her health. First, we wanted to determine if the decision to obtain a vaccination one season would be influenced by whether a person had obtained a vaccination the prior season or not. For each of the three years, a subject was categorized as vaccinated or unvaccinated and personal characteristics were compared based on the vaccination status. We also wanted to determine if the decision to receive a vaccination would be influenced by the person's utilization of outpatient or inpatient services for flu-like acute and chronic respiratory conditions [19] during the prior influenza season or interim season. A season was defined as an influenza season based on active influenza-surveillance data reported by Taiwan's CDC, usually from October of one year to the next year's April. The interim (non-epidemic) season usually lasted from May to September. Based on the total number of outpatient visits and hospitalizations, subjects were categorized into non-users, one-time users, and multiple users of medical services for these respiratory conditions each year. Our major outcome measurement was whether an older adult received an influenza vaccination or not during a specific influenza season. We recorded whether or not the person received a vaccination as well as when and at what facility he or she was vaccinated.

In addition to the threat-responsiveness variables, we analyzed our sample by age, gender, and number of chronic diseases. Subjects were categorized into four age groups: 65–69, 70–74, 75–79, and 80 years old or older. They were also categorized into how many chronic diseases they had, from 0 to more than three of any of the following chronic diseases: diabetes, chronic lung diseases, chronic kidney disease, chronic heart disease, cancer, and immunodeficiency [19-21], as WHO has specifically recommended that patients with these chronic diseases receive influenza vaccinations.

This study linked the outpatient claims with inpatient records, and the medical facility with region (northern, southern, central, or eastern) and hospital level (medical center, regional hospital, district hospital or clinic). In Taiwan, medical centers and regional hospitals have more beds and are more likely to have teaching accreditation, while district hospitals and clinics are smaller and less likely to have teaching accreditation. To analyze our data, we first used Chi-square tests to compare individual characteristics and influenza vaccination status at the national level among each year. Then, using multivariate logistic regression, we analyzed the influence of obtaining a prior influenza vaccination status and frequency of use of medical care services for flu-like respiratory conditions during a prior influenza season or during an interim (non-epidemic) season on the probability that the participants would receive an influenza vaccination the following influenza season. All variables found to be significant in our bivariate analyses were considered together in our model. We controlled for individual characteristics (age, gender, and the presence of WHO identified chronic diseases), the facility from which they routinely received medical care, the region in which the medical care facility was located, and the year of visit or vaccination. All statistical operations were performed using STATA 9.2 (College Station, Texas, USA).

## Results

### Older adult population

In total, we tracked the vaccination and utilization history of 23,023 older adults throughout the study period. The overall yearly vaccination rates for our sample were 38.6%, 44.3% and 39.3% for 2002, 2003, and 2004, respectively. As seen in Additional file 1, over sixty percent of those vaccinated were between 65 and 74 years old. The older the population, the smaller the proportion of people receiving vaccination (*p *< 0.001). A greater proportion of people with chronic diseases obtained vaccinations than those without the chronic diseases (*p *< 0.001). Regardless of vaccination status, most of these older adults resided in the northern Taiwan, followed by southern, central and eastern Taiwan (*p *< 0.001). Most sought medical care in clinics and district hospitals rather than medical centers and regional hospitals (*p *< 0.001). There was a less significant gender difference in those who were vaccinated and those who were not.

### Prior influenza vaccinations

We also compared previous threat-responsiveness related behaviors (prior vaccinations and previous visits to physicians for flu-like respiratory conditions) of those who received vaccination and those who did not (Additional file 1). As expected, we found a relationship between receiving a vaccination and having had a vaccination the prior year, with proportions increasing over time, ranging from 61.9% in 2002 to 79.6% in 2004 (*p *< 0.001). The opposite was true for those who had not previously received influenza vaccinations.

### Prior physician visits for flu-like respiratory conditions

We analyzed whether a person obtained a vaccination by the number of prior outpatient visits for flu-like respiratory conditions during a prior influenza season and during an interim season. We found that more than thirty percent of those who obtained vaccinations had previously made outpatient visits to physicians for acute or chronic respiratory conditions during the prior influenza season, with more than twenty percent having made two or more visits previously (Additional file 1). Over eighty percent of subjects who did *not *obtain vaccinations for one season had not made any outpatient visits for flu-like respiratory conditions during the prior influenza season (*p *< 0.001). We also analyzed association between being vaccinated and utilization during prior interim season. Those who had made outpatient visits to physicians during the interim season were found obtain vaccinations in significantly greater proportion than those who had sought similar care during the prior influenza season. More than forty percent of those obtaining vaccinations had previously made outpatient visits for flu-like respiratory conditions during the interim season (*p *< 0.001). In addition to outpatient visits, we also analyzed whether current vaccination status by the number of prior inpatient visits for flu-like respiratory conditions during prior influenza seasons and interim seasons. We found significant association between being previously admitted as inpatients for these conditions regardless of season and vaccination status.

### Regression analysis

We used multivariate logistic regression to study the relationship between receiving a current vaccination with prior influenza vaccination and number of previous visits to physicians for flu-like respiratory conditions, adjusting for age, gender, chronic diseases, the facility they routinely visited, the region the facilities were located and year of visit (Additional file 2). We found that those who had had received a vaccination in a prior influenza season were ten times more likely to receive another vaccination in the following influenza season than those who had not (OR = 10.22, 95% CI: 9.82–10.64). We also found that the greater the frequency of prior physician visits for flu-like respiratory conditions, the greater the likelihood that one would decide to be vaccinated. Visits during prior interim season exerted a stronger positive influence than prior influenza season on this likelihood (OR = 1.59, 95% CI: 1.46–1.73 vs. OR = 1.11 95% CI: 1.01–1.22, respectively). Being admitted as an inpatient did not have a similar effect, regardless of whether the subjects have been admitted during a prior influenza season or interim season. Compared to those usually went to larger medical centers, significantly larger proportion of older patients were more likely to have vaccination at smaller medical facilities, clinics (OR = 4.94. 95%CI: 4.54–5.37) and district hospitals (OR = 5.54, 95%CI: 5.05–6.08) (*p *< 0.01).

In our multivariate logistic regression models, we also analyzed the possible effect of age, gender and number of chronic diseases on the likelihood that one would obtain a vaccination (Additional file 2). Compared to our youngest age group, those between 70 and 74 years old were more likely and those 80 years old or older were less likely to have influenza vaccinations (OR = 1.23, 95% CI: 1.17–1.30 and OR = 0.68, 95%CI: 0.64–0.72). Men were more likely than women to obtain influenza vaccinations (OR = 1.09, 95%CI: 1.05–1.13). The greater the number of chronic diseases a patient had, the greater the likelihood that he or she would obtain a vaccination. Compared to those who had no chronic disease, ORs range from 1.68 to 1.91. Those living in central and southern Taiwan were significantly more likely to obtain influenza vaccinations than those living in northern Taiwan (*p *< 0.01).

## Discussion

This study found that prior influenza vaccination, number of prior outpatient visits for flu-like respiratory conditions, especially during the interim season, to be the most significant predictors of whether an older adult would decide to obtain an influenza vaccination. In other words, perceived threat is a significant predictor of vaccination status among the older adults.

Adjusting for covariates, our multivariate logistic regression revealed that older adults who received influenza vaccinations during the prior influenza season to be significantly more likely to obtain another vaccination the following season, supporting our hypothesis. This trend is consistent with findings of studies done in Hong Kong and Spain [22,23], though ours found a much greater influence from this factor. Although the mechanism underlying this phenomenon needs further investigation, the vaccination rate among the older adults might be improved by programs promoting first time influenza vaccinations among the older adults.

We also found frequency of outpatient visits for flu-like respiratory conditions during the previous influenza season and interim season to have had a significantly positive effect of the likelihood that people would obtain a vaccination. Some studies with similar findings have attributed this increase in vaccination rate with the increased attention and physician recommendations the patients receive during these visits [2,23-25]. Since influenza is well-known to exacerbate respiratory diseases, increase related hospitalization and morbidity rates [26] as well as carry some risk of mortality [27], the threat older adults perceive while being treated for respiratory conditions predispose them to obtaining influenza vaccinations. Therefore, our study used frequency of utilization of such services as proxy representing a participant's threat-responsiveness [14,15,17]. Although it might be difficult to distinguish between the effect of physician recommendation and threat-responsiveness, we also observed the time that these services were used also exerted a significant effect on the decision to obtain a vaccination. Patients using these services during the prior interim season, the season closest to influenza-vaccination season, were more likely than those using them during the prior influenza season to obtain a vaccination, suggesting that our subjects responded to more immediate disease threats, as was found by one other previous study in US [14].

Similar to other studies [2,10,23,28], the patients who had more chronic diseases in our study were more likely to obtain influenza vaccinations than those who did not. Our study also found that patients between 70 to 74 years old to be significantly more likely than those between 65 and 69 year old to obtain influenza vaccinations (*p *< 0.01), similar to reports from Canada, Spain, and Hong Kong [10,23-25,28,29]. Surprisingly, our oldest group (age 80 or over) were significantly less likely than our youngest group to obtain vaccinations (*p *< 0.01). It is not known why there is a sudden decrease in likelihood among the oldest. It may be related to decreased ability to get around, less support, or greater fear of their own fragility. However, because the oldest are most vulnerable during the influenza season, more effort needs to be devoted to encouraging their participation in the prevention program. In addition, older men were more likely than older women to obtain influenza vaccination in our study (*p *< 0.01), a finding not always consistent with previous studies regarding the influence of gender of this decision [30-32]. This difference might be a result of racial/ethnicity, differences in age, differences in chronic diseases, or even differences level of apprehension of getting along independently outside, though these hypotheses require further study to confirm.

This study has several possible limitations. First, claims data cannot provide detailed information regarding the socio-economic level of the older subjects. The limitation may not be as serious as it seems, however, because Taiwan's universal insurance program, which covers most of the health care costs of over 98% of its population, effectively reduces most of the socio-economic barriers to health care found in countries that do not provide insurance to their citizens. Another possible limitation with using claims records is that they lack lifestyle and health behavior data. Future studies might want to link individual socio-economic status, lifestyle, and health behavior data collected from surveys with medical claims records to do a more comprehensive analysis.

The use of insurance claims, however, also has several advantages. One advantage is that randomly chosen claims records from a universal insurance system that covers almost 100% a country's population are nationally representative, making it possible to more accurately track medical care use and individual medical care history within the whole health care delivery system. Previous studies directly exploring key factors associated with decisions to obtain influenza vaccinations have focused more on individual demographic characteristics, health status, socio-economic status or health insurance coverage [9,23,27,29,34,35]. Many of those studies were conducted using surveys, which may, due the self-reporting they involve, have had some problems with such limitations as recall bias [23,35,36]. Recall bias may be especially relevant when surveying older adults, some of whom may have problems remembering exact times. Our use of claims records helps avoid potential flaws related to recall.

The influenza vaccine coverage rates for older adults in Taiwan observed in this study (38.6%, 44.3% and 39.3% for 2002, 2003, and 2004) were not as high as those observed in other developed countries. For similar study periods, the influenza vaccine coverage for older adults in US was 65%, in Canada 62%, and in Australia 79% [37-39]. Meanwhile, a report comparing influenza vaccine coverage rates for those aged 65 years and older in European countries indicated that most of the studied countries had vaccine coverage rates higher than 50% (e.g., Netherlands 74%, UK 73%, Spain 69%, France 67%, and Germany 55%) [40]. However, when directly comparing vaccine coverage rates among different countries, differences in health care delivery system, health insurance coverage, social/cultural factors, and demographic characteristics need to be taken into consideration. Taiwan has universal health care coverage increases access to care and reduces economic barriers. Nevertheless, a comparison of countries reveals that there is enough room for further vaccination rate increases among older adults in Taiwan.

## Conclusion

Free vaccination coverage does not guarantee high vaccination rates. Continual ongoing public health interventions may be necessary in order to generate optimal vaccination rates. While increased access to vaccinations may improve vaccination rates and reduce infections during epidemics of infectious disease, so may individual responses to disease threats. Our results show that prior influenza vaccination, frequency of prior outpatient visits for flu-like respiratory conditions, particularly during the interim season (closer to the following epidemic season) significantly predict the vaccination status of an older adult. Perceived threat is a significant predictor of vaccination status among the older adults. These findings can be used to help design public health policies and campaigns to increase the vaccination rate of this vulnerable group of citizens. In particular, older adults who never had influenza vaccinations can be identified, educated and encouraged to participate in this important program.

## Competing interests

The authors declare that they have no competing interests.

## Authors' contributions

YCL designed the project, contributed to the data acquisition, data analysis and interpretation, supervised the study development, and wrote the manuscript. CML contributed to data analysis and organized the draft of the manuscript. All authors read and approved the final manuscript and additional files

## Pre-publication history

The pre-publication history for this paper can be accessed here:



## Supplementary Material

Additional file 1 **Table 1**. Characteristics of the study sample by influenza vaccination status and years. Click here for file

Additional file 2 **Table 2**. Adjusted odds ratios (ORs) and 95% confidence intervals (95% CIs) for influenza vaccination associated with prior influenza vaccination status and related medical care utilizations. Click here for file
