# Supplementary material for: Threat-responsiveness and the decision to obtain free influenza vaccinations among the older adults in Taiwan
Source: BMC Public Health. 2009 Jul 31;9:275. doi: 10.1186/1471-2458-9-275 (PMC2734847; doi:10.1186/1471-2458-9-275)
Supplement: Additional file 1 — Table 1. Characteristics of the study sample by influenza vaccination status and years. [file 1471-2458-9-275-S1.doc]

|  | 2002 | | | 2003 | | | 2004 | | |
| --- | --- | --- | --- | --- | --- | --- | --- | --- | --- |
| Characteristics | Unvaccinated  (*N =14,133*) | Vaccinated  (*N=8,890*) | *P*-value^*^ | Unvaccinated  (*N=12,829*) | Vaccinated  (*N=10,194*) | *P*-value^*^ | Unvaccinated  (*N=13,979*) | Vaccinated  (*N=9,044*) | *P*-value^*^ |
| Age |  |  |  |  |  |  |  |  |  |
| 65-69 | 48.2% | 32.6% | <0.001 | 41.5% | 32.3% | <0.001 | 33.3% | 31.0% | <0.001 |
| 70-74 | 18.8% | 32.5% |  | 19.4% | 30.3% |  | 21.6% | 30.4% |  |
| 75-79 | 14.8% | 20.1% |  | 16.5% | 21.6% |  | 18.7% | 21.8% |  |
| $\geq$80 | 18.3% | 14.8% |  | 22.6% | 15.8% |  | 26.4% | 16.9% |  |
| Gender |  |  |  |  |  |  |  |  |  |
| Women | 49.4% | 47.9% | 0.028 | 49.2% | 48.4% | 0.254 | 49.1% | 48.5% | 0.383 |
| Men | 50.6% | 52.1% |  | 50.8% | 51.6% |  | 50.9% | 51.5% |  |
| # of chronic diseases |  |  |  |  |  |  |  |  |  |
| 0 | 23.1% | 8.8% | <0.001 | 23.1% | 10.6% | <0.001 | 21.5% | 11.5% | <0.001 |
| 1 | 30.7% | 25.9% |  | 29.9% | 27.4% |  | 28.6% | 29.2% |  |
| 2 | 28.9% | 37.3% |  | 28.8% | 36.4% |  | 30.0% | 35.5% |  |
| $\geq$3 | 17.4% | 28.0% |  | 18.2% | 25.6% |  | 19.9% | 23.9% |  |
| Received flu shot in last flu season |  |  |  |  |  |  |  |  |  |
| No | 86.1% | 38.1% | <0.001 | 85.4% | 31.2% | <0.001 | 78.6% | 20.4% | <0.001 |
| Yes | 14.0% | 61.9% |  | 14.7% | 68.8% |  | 21.5% | 79.6% |  |

Table 1: Characteristics of the study sample by influenza vaccination status and years

Table 1. Characteristics of the study sample by influenza vaccination status and years (cont.)

|  | 2002 | | | 2003 | | | 2004 | | |
| --- | --- | --- | --- | --- | --- | --- | --- | --- | --- |
| Characteristics | Unvaccinated  (*N=14,133*) | Vaccinated  (*N=8,890*) | *P*-value^*^ | Unvaccinated  (*N=12,829*) | Vaccinated  (*N=10,194*) | *P*-value^*^ | Unvaccinated  (*N=13,979*) | Vaccinated  (*N=9,044*) | *P*-value^*^ |
| # of outpatient visits in last flu season |  |  |  |  |  |  |  |  |  |
| 0 | 83.2% | 66.4% | <0.001 | 82.4% | 67.1% | <0.001 | 81.4% | 68.6% | <0.001 |
| 1 | 6.7% | 12.1% |  | 6.8% | 12.0% |  | 6.9% | 11.4% |  |
| $\geq$2 | 10.1% | 22.6% |  | 10.9% | 21.0% |  | 11.6% | 20.0% |  |
| # of outpatient visits in interim season |  |  |  |  |  |  |  |  |  |
| 0 | 74.4% | 52.2% | <0.001 | 74.7% | 54.5% | <0.001 | 72.7% | 55.8% | <0.001 |
| 1 | 8.8% | 13.7% |  | 8.3% | 13.7% |  | 8.9% | 14.1% |  |
| $\geq$2 | 16.8% | 34.1% |  | 17.0% | 31.9% |  | 18.4% | 30.1% |  |
| # of hospitalizations in last flu season |  |  |  |  |  |  |  |  |  |
| 0 | 97.3% | 97.8% | 0.006 | 96.6% | 97.9% | <0.001 | 96.7% | 97.9% | <0.001 |
| 1 | 1.9% | 1.8% |  | 2.4% | 1.8% |  | 2.3 % | 1.7% |  |
| $\geq$2 | 0.8% | 0.4% |  | 1.0% | 0.3% |  | 1.0% | 0.4% |  |

Table 1. Characteristics of the study sample by influenza vaccination status and years (cont.)

|  | 2002 | | | 2003 | | | 2004 | | |
| --- | --- | --- | --- | --- | --- | --- | --- | --- | --- |
| Characteristics | Unvaccinated  (*N=14,133*) | Vaccinated  (*N=8,890*) | *P*-value^*^ | Unvaccinated  (*N=12,829*) | Vaccinated  (*N=10,194*) | *P*-value^*^ | Unvaccinated  (*N=13,979*) | Vaccinated  (*N=9,044*) | *P*-value^*^ |
| # of hospitalizations in interim season |  |  |  |  |  |  |  |  |  |
| 0 | 94.4% | 95.7% | <0.001 | 93.4% | 95.9% | <0.001 | 93.5% | 96.0% | <0.001 |
| 1 | 3.6% | 3.0% |  | 4.3% | 3.2% |  | 4.1% | 2.9% |  |
| $\geq$2 | 2.0% | 1.3% |  | 2.4% | 0.9% |  | 2.4% | 1.1% |  |
| Most common place for medical care |  |  |  |  |  |  |  |  |  |
| Medical center | 14.3% | 2.9% | <0.001 | 14.2% | 3.7% | <0.001 | 14.3% | 3.2% | <0.001 |
| Regional hospital | 14.3% | 7.1% |  | 14.3% | 7.9% |  | 14.2% | 7.9% |  |
| District hospital | 13.6% | 19.1% |  | 14.3% | 20.0% |  | 14.6% | 18.2% |  |
| Clinic | 57.8% | 71.0% |  | 57.2% | 68.5% |  | 57.0% | 70.8% |  |
| Region |  |  |  |  |  |  |  |  |  |
| North | 45.6% | 34.5% | <0.001 | 44.7% | 37.0% | <0.001 | 45.0% | 35.7% | <0.001 |
| Central | 22.3% | 29.0% |  | 22.1% | 28.6% |  | 22.1% | 29.0% |  |
| South | 28.0% | 32.5% |  | 29.0% | 30.6% |  | 28.6% | 31.4% |  |
| East | 4.2% | 4.0% |  | 4.3% | 3.9% |  | 4.3% | 3.9% |  |
