# Supplementary material for: Threat-responsiveness and the decision to obtain free influenza vaccinations among the older adults in Taiwan
Source: BMC Public Health. 2009 Jul 31;9:275. doi: 10.1186/1471-2458-9-275 (PMC2734847; doi:10.1186/1471-2458-9-275)
Supplement: Additional file 2 — Table 2. Adjusted odds ratios (ORs) and 95% confidence intervals (95% CIs) for influenza vaccination associated with prior influenza vaccination status and related medical care utilizations. [file 1471-2458-9-275-S2.doc]

Table 2: Adjusted odds ratios (ORs) and 95% confidence intervals (95% CIs) for influenza vaccination associated with prior influenza vaccination status and related medical care utilizations

| Variables | OR | 95% CI | *P*-value |
| --- | --- | --- | --- |
| Received flu shot in last flu season |  |  |  |
| No | 1.00 |  |  |
| Yes | 10.22 | 9.82-10.64 | <0.001 |
| Number of outpatient visits in last flu season |  |  |  |
| 0 | 1.00 |  |  |
| 1 | 1.03 | 0.94-1.13 | 0.527 |
| $\geq$2 | 1.11 | 1.01-1.22 | 0.034 |
| Number of outpatient visits in interim season |  |  |  |
| 0 | 1.00 |  |  |
| 1 | 1.51 | 1.40-1.63 | <0.001 |
| $\geq$2 | 1.59 | 1.46-1.73 | <0.001 |
| Number of hospitalization in last flu season |  |  |  |
| 0 | 1.00 |  |  |
| 1 | 1.06 | 0.87-1.30 | 0.549 |
| $\geq$2 | 0.75 | 0.52-1.07 | 0.114 |
| Number of hospitalization in interim season |  |  |  |
| 0 | 1.00 |  |  |
| 1 | 0.46 | 0.40-0.53 | <0.001 |
| $\geq$2 | 0.28 | 0.22-0.36 | <0.001 |
| Common place for medical care |  |  |  |
| Medical center | 1.00 |  |  |
| Regional hosp | 2.48 | 2.25-2.74 | <0.001 |
| District hosp | 5.54 | 5.05-6.08 | <0.001 |
| Clinic | 4.94 | 4.54-5.37 | <0.001 |
| Age |  |  |  |
| 65-69 | 1.00 |  |  |
| 70-74 | 1.23 | 1.17-1.30 | <0.001 |
| 75-79 | 1.02 | 0.97-1.08 | 0.478 |
| $\geq$80 | 0.68 | 0.64-0.72 | <0.001 |
| Gender |  |  |  |
| Women | 1.00 |  |  |
| Men | 1.09 | 1.05-1.13 | <0.001 |

Table 2: Adjusted odds ratios (ORs) and 95% confidence intervals (95% CIs) for influenza vaccination associated with prior influenza vaccination status and related medical care utilizations (cont.)

| Variables | OR | 95% CI | *P*-value |
| --- | --- | --- | --- |
| Number of chronic diseases |  |  |  |
| 0 | 1.00 |  |  |
| 1 | 1.68 | 1.59-1.78 | <0.001 |
| 2 | 1.89 | 1.79-2.00 | <0.001 |
| $\geq$3 | 1.91 | 1.79-2.04 | <0.001 |
| Region |  |  |  |
| North | 1.00 |  | <0.001 |
| Central | 1.33 | 1.27-1.40 | <0.001 |
| South | 1.14 | 1.09-1.20 | <0.001 |
| East | 0.97 | 0.88-1.07 | 0.571 |
| Year |  |  |  |
| 2002 | 1.00 |  |  |
| 2003 | 1.18 | 1.13-1.24 | <0.001 |
| 2004 | 0.74 | 0.71-0.78 | <0.001 |

Note: 1. Outpatient visits and hospitalizations were associated with flu-like respiratory conditions.

2. Chronic diseases include diabetes, chronic lung diseases, chronic kidney disease, chronic heart disease, cancer, and immunodeficiency.
